# Etiologies of Orthopedic Implant Removal Among Patients Who Underwent Orthopedic Fixation Surgeries in King Abdulaziz Medical City

**DOI:** 10.7759/cureus.43809

**Published:** 2023-08-20

**Authors:** Wazzan ALJuhani, Mohammed H Almusallam, Mohammad S Almosa, Musaad M Bin Dukhi, Abdulaziz M Bin Akrish, Soliman A Alaraidh, Abdullah Alanazi

**Affiliations:** 1 Department of Surgery, Ministry of the National Guard - Health Affairs, Riyadh, SAU; 2 Department of Surgery, King Abdullah International Medical Research Center, Riyadh, SAU; 3 Department of Surgery, King Saud Bin Abdulaziz University for Health Sciences, Riyadh, SAU; 4 College of Medicine, King Saud Bin Abdulaziz University for Health Sciences, Riyadh, SAU

**Keywords:** hardware removal, hardware implantation, complications, reason for removal, hardware

## Abstract

Background: Hardware removal surgeries are considerably common. However, the justifications for these surgeries are debatable. The indications may vary from pain, surgical site infection, or failure of osteosynthesis. Additionally, the surgery can be performed without medical indication. Following these surgeries, many complications can occur. Therefore, surgeons and patients should be aware of the appropriate indications and have realistic expectations of the risks and benefits of implant removal.

Methods: This was a retrospective chart review study. We included all patients aged 17-86 years who underwent hardware removal surgery in the orthopedic surgery department of King Abdulaziz Medical City, Riyadh, Saudi Arabia, from 2010 to 2019. All relevant data, such as demographic characteristics, location and type of hardware, indication for hardware removal, duration between insertion and removal, and complications after removal were recorded and analyzed.

Results: A total of 244 medical records were reviewed with a mean patient age of 34 years. Out of the total, 190 patients (77.9%) were male. The femur was the most common location of hardware removal, in 50 cases (20.58%). Plates and screws were the most common type of implants (40.2%). Most patients underwent hardware implantation because of fractures (89.3%). In total, 119 patients (48.8%) had the hardware removed electively. Only 29 patients (12%) reported postoperative complications; 190 patients (77.9%) were advised by their surgeon against hardware removal.

Conclusion: Hardware removal procedures are commonly performed worldwide for various reasons, including infection, pain, and loosening. In this study, we have outlined the reasons for implant removal, the locations, complications, and the rationale behind this surgery. We have provided a new decision-making assessment, aimed at the general population and surgeons alike, that will help patients better comprehend the complications and risks associated with this elective surgery.

## Introduction

Hardware removal is considered to be one of the most commonly performed surgeries in the modern world. There is, however, a great deal of debate regarding the justification and indications for these surgical procedures [1]. Hardware removal surgeries are indicated when a patient suffers from surgical site infection, soft tissue damage, or failure of osteosynthesis [2]. Furthermore, a retrospective review study conducted at a German level-1 trauma center that included 332 patients who underwent orthopedic hardware removal procedures found that 31% underwent the procedure because of pain and 24% due to foreign body sensation [3]. Hardware removal surgeries are sometimes performed based only on patient preferences without well-defined indications. For example, labor workers who may require a greater range of motion or individuals who may have misconceptions regarding the various types of hardware implants may undergo surgery and suffer complications as a result.

The presence of infection is one of the most common reasons for patients to undergo hardware removal surgery; it may also be a determinant of failure following open reduction and internal fixation procedures (ORIFs) [4]. It was reported that the highest rates of orthopedic hardware removal complications occurred because of impaired wound healing, followed by infection, comprising 36% and 21%, respectively [3]. 

Furthermore, liver disease, rheumatoid arthritis, and diabetes are predictors of infection associated with hardware implants after ORIF [4]. Broken hardware is also an indication for surgery. Hardware damage is often discovered on pre-operative radiographs but can also be accidentally discovered during surgery [5]. Overlapping hardware can obscure the visualization of adjacent structures on pre-operative radiographs [6]. Furthermore, special equipment is needed to remove stripped screws or broken intramedullary nails. In the case of broken screws, the screw heads can be easily loosened or removed, while the distal end of the screw remains embedded in the bone, which may increase the operating time and complexity [7].

Interestingly, it is often overlooked how cold weather affects recovery following hardware removal surgery. A study was conducted on the effect of cold weather on 100 patients who underwent orthopedic fixation surgery and assessed whether occupational status, age, and sex had an effect on pain [8]. The study showed that 49% of the patients experienced pain during winter, whereas 51% experienced no pain. Moreover, 46% of their patients had expressed that pain persisted in the summer with the use of cold air conditioning; this observation suggests a significant correlation between cold weather and pain in these patients. In terms of pain severity, 20% of the patients had mild pain, and 29% had serious pain. Occupational status, age, and sex did not show any relation in terms of pain perception [8].

Despite the fact that orthopedic hardware removal surgeries have some indications, many complications may occur as a result of these procedures. Among these complications, delayed wound healing, infections, nerve injury, unexpected reoperations, and bone fractures are commonly observed. [9]. The aforementioned German study has also found that out of the 332 patients who underwent hardware removal surgery, 7% had a refracture, and 3% had observed keloid development and bleeding. It was also shown that there is a correlation between the site of the implanted hardware and the occurrence of these complications [3].

Currently, there is a scarcity of published articles on the topic of hardware removal surgery in the Kingdom of Saudi Arabia. Thus, our goal was to conduct this study locally. Although hardware removal surgery is commonly performed around the world, clinical indications for hardware removal are still not fully established [10]. Surgeons and patients should be aware of the appropriate indications for implant removal and establish realistic expectations regarding the risks and benefits involved. Therefore, the aim of our study was to assess the causes and reasons for hardware removal among orthopedic patients.

## Materials and methods

Study design, area, and settings

King Abdullah International Medical Research Center (KAIMRC) issued the approval of this study with study number SP20/338/R. This study was conducted in the orthopedic surgery department of King Abdulaziz Medical City, Riyadh, Saudi Arabia. We retrospectively reviewed the records of all patients who underwent hardware removal surgery between January 2016 and January 2019. A cross-sectional chart review was conducted to determine the prevalence of and reasons for hardware removal surgeries.

Identification of study participants

All patients aged 17-86 years old who underwent hardware removal surgeries between 2016 and 2019 were included. This study had no exclusion criteria. Raosoft software (Raosoft Inc., Seattle, WA) was used to calculate the sample size. The estimated sample size was 244. We used a non-probability consecutive sampling technique for all the patients who underwent hardware removal surgeries.

Data collection process

The research group members collected the data using patient medical records from the BestCare system. In this study, categorical (causes of hardware removal, sex, nationality, location of the hardware, any infection or pain, blood loss, refracture after hardware removal) and numerical (BMI and age) variables were examined.

Statistical analysis

All the required data were entered into an Excel data collection sheet and then transferred to SPSS version 25 (IBM Corp., Armonk, NY), which was used for data analysis. Categorical data were reported as frequencies and percentages, while numerical data were reported as mean ± standard deviation. The chi-square test was used to compare categorical data. ANOVA was used to compare numerical data. The prevalence of hardware removal surgeries was estimated at a confidence level of 95%. Statistical significance was set at p < 0.05. Since this study involved a chart review without patient identification information, informed consent was not required. Conversely, a serial number was assigned to each patient for identification purposes. To secure patient data, confidentiality practices, such as encryption and password protection, were used. Identifiable data about the patients were not obtained. During and after the study, only members of the research team had access to the data.

## Results

A total of 244 medical records of patients who underwent hardware removal surgery were reviewed. Demographic data showed that the patients had a mean age of 34 years (range, 17-86 years) (Table 1). More than three-quarters of the patients (190, 77.9%) were men. Most patients were from Saudi Arabia (232 patients, 95.1%). In terms of employment, 65 patients (26.6%) were unemployed, 53 (21.7%) were students, and 63 (25.8%) were employed in the military (Table 1). With regard to marital status, 141 patients (58.6%) were married. The number of smokers among the patients was 65 (26.6%). There were only 16 patients (6.6%) with allergies. Most of the patients (73, 30.3%) had a normal body mass index (18-25), while 70 patients (29%) were classified as overweight; the mean BMI for all patients was 27.77 kg/m2. Most patients (190, 77.9%) were advised by their doctor not to remove their hardware implants, while only 54 (22.1%) were advised to undergo surgery.

**Table 1:** Demographic characteristics of patients who underwent hardware surgery Abbreviations: National Guard Health Affairs (NGHA), not available (N/A)

Characteristics	Frequency	Percentage
Age Group (years)		
17–30	129	52.9
31–44	70	28.7
45–58	29	11.9
59–72	12	4.9
73–86	4	1.6
Total	244	100
Jobs		
Unemployed	65	26.6
Soldier	63	25.8
Student	53	21.7
Others (including nurses, civil workers, homemakers, retired, clerks, teachers, NGHA staff, and engineers)	51	21
Total (excluding N/A)	232	95.1
N/A	12	4.9

**Figure 1:**
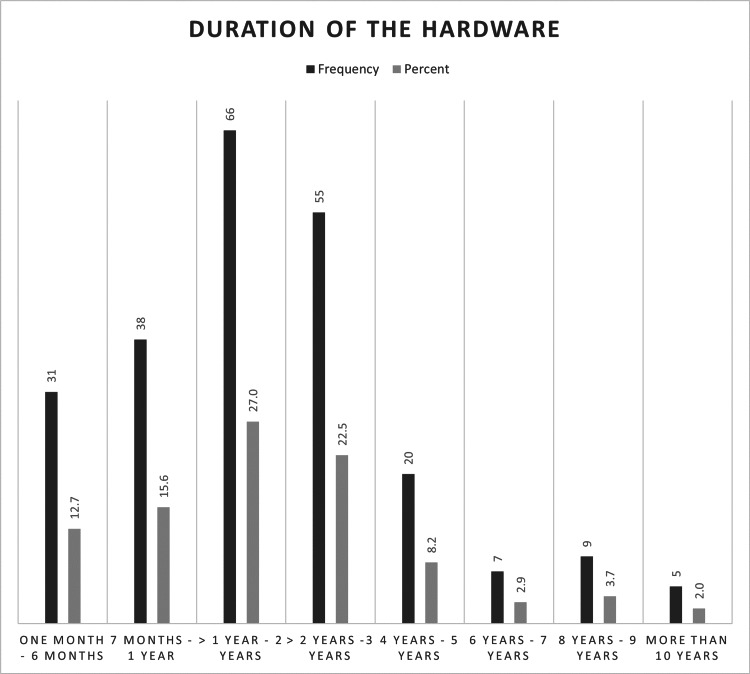
Duration of the hardware This figure illustrates the time between the insertion and removal of hardware reported as frequencies and percentages (n=231).

In most cases, patients had their implants for 1-2 years (27%) before presenting for hardware removal. However, out of the 244 patients, 13 had no records regarding the duration of their implant (Figure 1). Implants were most frequently removed from the femur (50, 20.58%) and ankle (41, 16.8%), followed by the tibia (31, 12.7%). Table 2 outlines the relationship between the location of the hardware implant and the reason for its removal.

**Table 2:** Location of the hardware and the reasons for its removal

		Reason For Removal						Total Count
		Elective removal/fracture healed	Pain	Military	Appearance or foreign body sensation	Hardware issues or complications	Refracture	
Location of Hardware	Femur	32	9	3	0	3	3	50 (20.58%)
	Ankle	24	7	1	1	3	5	41 (16.88%)
	Tibia	14	8	4	1	3	1	31 (12.76%)
	Humerus	7	3	1	1	4	2	18 (7.4%)
	Clavicle	7	5	0	0	3	2	17 (7%)
	Foot	5	8	0	1	2	0	16 (6.58%)
	Others (including the elbow, forearm, wrist, hand, shoulder, pelvis, patella, face, fibula, and spine)	30	20	1	6	11	2	70 (28.8%)
Total Count		119	60	10	10	29	15	243

**Figure 2:**
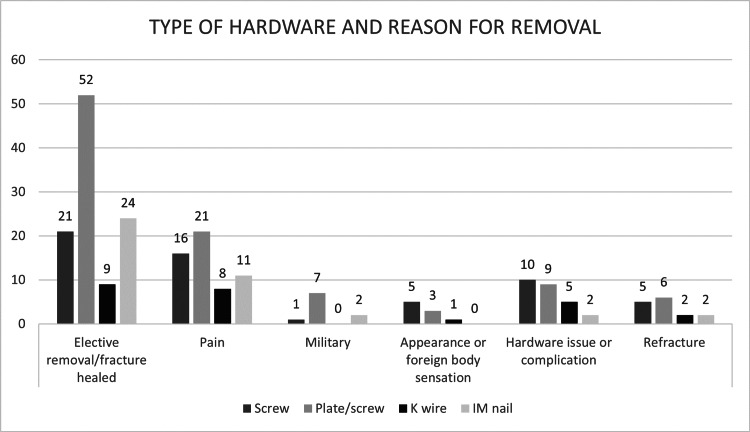
Type of hardware and reason for removal This figure shows and compares the types of implants used and the reasons for their removal reported as frequencies. Abbreviations: intramedullary nail (IM nail), Kirschner wire (K-wire).

A total of 98 patients (40.2%) had both plates and screws implanted; these were the most common types of hardware implants. Conversely, 58 patients (23.8%) had screws as implants (Table 3). Furthermore, Figure 2 illustrates the relationship between the type of implant used and the reason for its removal.

**Table 3:** Type of hardware removed Abbreviations: Intramedullary nail (IM nail), Kirschner wire (K-wire)

Type of Hardware	Frequency	Percentage
Plate/screw	98	40.2
Screw	58	23.8
IM nail	41	16.8
K-wire	25	10.2
Others (Including pins, rods, staplers, Hunter rods, tension wires)	21	8.6
Total (excluding N/A)	243	99.6
N/A	1	0.4
Total	244	100

Most patients underwent hardware implantation surgeries due to fractures (218, 89.3%), followed by congenital deformities (12, 4.9%), and only eight patients (3.3%) had implant infections. Prior to surgery, 38 patients (15.6%) had orthopedic comorbidities, the majority of which was osteoarthritis, as noted in 10 patients (4.1%).

Out of the 244 patients, 119 (48.8%) had their implants removed electively. Conversely, 60 (24.6%) had their implanted hardware removed due to pain, and 15 patients (6.1%) because of a refracture. Among the 190 male patients, 10 (4.8%) requested the removal of their hardware implants because of military regulations.

The study showed that 11 (9%) out of the 119 patients who had their hardware implants removed electively had post-operative complications. Similarly, only one of the nine patients who had the hardware removed due to military regulations experienced post-operative complications. Table 4 illustrates the postoperative complications after hardware removal. Altogether, 214 (88%) out of all patients had no complications after hardware removal surgery, while a total of 16 patients (6.6%) reported post-operative pain and eight (3.2%) experienced refractures, arthritis, muscle spasms, or stiffness, with each complication presenting in two patients. Five patients (2%) developed either keloid scars, wound dehiscence, nerve damage, avascular necrosis, or infection.

**Table 4:** Comparison between the reasons for hardware removal and post-operative complications after removal

		Reasons For Removal						Total
		Elective removal/fracture healed	Pain	Military regulations	Appearance or foreign body sensations	Hardware issues or complications	Refracture	
Post-operative complications after removal	No	108	50	9	10	24	13	214
		90.70%	83.33%	90%	100%	82.75%	86.70%	88%
	Yes	11	10	1	0	5	2	29
		9.30%	16.70%	10%	0%	17.25%	13.30%	12%
Total		119 (48.97%)	60 (24.69%)	10 (4.12%)	10 (4.12%)	29 (11.93%)	15 (6.17%)	243

## Discussion

The main findings of our study are the reasons for hardware removal and the postoperative complications. In our patient sample, most hardware removal surgeries were performed as elective procedures (48%), and most of these surgeries were not recommended by their surgeons. The German study that included 332 patients indicated that 30% of their patients underwent hardware removal surgeries based on their personal preference, which was considered a high percentage [3]. Even though the percentage of patients who underwent hardware removal surgeries based on personal preference was high, it was significantly higher in our study. This higher percentage is attributed to the low awareness of hardware safety among our patients. Moreover, as a governmental hospital in Saudi Arabia, the price of the surgery is paid for, making a high number of patients attempt to undergo the surgery electively, as they do not bear expenses. Nevertheless, 22.1% of the surgeries were performed on the advice of a physician due to various medical indications. Despite the fact that 10 (5.2%) of the 190 male patients reported that they underwent hardware removal surgery due to military regulations, this figure may not reflect the actual number of military personnel who underwent the surgery to meet their job requirements. 

In this study, 12% of our population, which is 244 patients, had postoperative complications. Pain was considered the most common complication, accounting for 6.6% of all cases. Conversely, 10% of the patients in the aforementioned German level-1 trauma center retrospective review study experienced postoperative complications mainly due to poor wound healing. In their study, they depended on patients reporting poor wound healing, whereas in our study, we depended on the wound care assessment unit [3]. In contrast, a study from the University of Ilorin Teaching Hospital reported infection as the most common postoperative complication (57%) and related it to a higher level of pre-operative infection of the wound or hardware [11].

In the presented study that was conducted in a National Guard Hospital, a government-funded health system, 25.8% of the patients who had their hardware removed were military personnel. Many of the National Guard Hospital patients are soldiers, and hence they decide to take the implant out due to their job requirements or even to be enrolled in the military. This fact might have influenced the reasons for hardware removal, as elective removal by the patient's wishes was the most common choice. Since military personnel were the most commonly operated patients in this study, these results may not be generalizable to the entire population.

A total of 50 patients (20.58%) were operated on at the femur in our study, making it the most operated site in our cohort. Moreover, other studies report similar findings, with hardware removals from the lower extremities, such as the femur and tibia, being the most prevalent [12,13]. In a different study [14], the ankle joint was the most common location for surgery, whereas, in our study, it ranked second (41 patients, 16.8%).

Our cohort was predominantly male, which is consistent with other studies [11,13]. In a previous study performed on 83 patients who had undergone implant removal, 85.5% were male, which is consistent with our finding (77.9%) [12]. Furthermore, most of our patients were between 17 and 30 years of age (52%). In our study, the patient's age group ranged from 17-86 years, with a mean of 34, which is congruous with the previous study where the patient sample ranged from 20-78 years, with a mean of 38 years [12].

The mean BMI calculated for the collected sample was 27.77 kg/m2, which is classified as overweight. In a study that compared the relationship between high body weight and bone health, one pound of body weight could place a high burden on each knee (about four to six pounds) [15]. The adverse effects of high body weight on surgical outcomes and complications are also significant. These complications include higher rates of prosthesis failure or loosening as well as infection of the hardware implant [15].

Out of the 244 patients sampled, 38 (15.6%) had concurrent orthopedic comorbidities such as osteoporosis, osteopenia, and rheumatoid arthritis. However, the most common orthopedic comorbidity in our cohort was osteoarthritis, which was present in 10 out of the 38 patients. In a study that was conducted to assess complications and outcomes of osteoarthritic knees following a medial opening wedge high tibial osteotomy (MOWHTO) using a locking plate, complications occurred in 29.3% of patients who underwent the procedure. Some of the described complications included hardware irritation (1.2%), hardware failure without associated symptoms (0.6%), and hardware failure with associated symptoms (0.6%) [16]. Nonetheless, there is still a gap in understanding whether diseases that affect the bone would have a different implant outcome compared to healthy bone. Thus, further research is warranted to determine the relationship between orthopedic comorbidities, hardware effectiveness, and complications.

Our study had a few limitations. Firstly, our study design was cross-sectional. Secondly, future studies should be extended to include more centers, as the National Guard Health Affairs patients are mostly military personnel, and the sample should be broadened in order to provide a clearer picture and eliminate potential biases.

## Conclusions

This is the first study that addresses the reasons for hardware removal surgeries that have been conducted in Saudi Arabia. As previously mentioned, we can observe the continuous issue of elective hardware removal surgeries, which puts the patient at risk of multiple complications. In this study, we have outlined the reasons for implant removal, the locations, complications, and some of the rationales behind performing this surgery. We have demonstrated that not all hardware removal procedures are due to patients’ fear or ignorance of hardware implants; rather, some procedures are due to pain and other medical indications. We have provided a new decision-making assessment aimed at the general population and surgeons alike that will help patients better comprehend the complications and dangers associated with this elective surgery.
